# PDE4 Phosphodiesterases in Cardiovascular Diseases: Key Pathophysiological Players and Potential Therapeutic Targets

**DOI:** 10.3390/ijms242317017

**Published:** 2023-11-30

**Authors:** Lídia Puertas-Umbert, Judith Alonso, Leif Hove-Madsen, José Martínez-González, Cristina Rodríguez

**Affiliations:** 1 Institut de Recerca Sant Pau (IR SANT PAU), 08041 Barcelona, Spain; lpuertas@santpau.cat (L.P.-U.); judith.alonso@iibb.csic.es (J.A.); leif.hove@iibb.csic.es (L.H.-M.); 2 CIBER de Enfermedades Cardiovasculares, Instituto de Salud Carlos III, 28029 Madrid, Spain; 3 Instituto de Investigaciones Biomédicas de Barcelona-Consejo Superior de Investigaciones Científicas (IIBB-CSIC), 08036 Barcelona, Spain

**Keywords:** PDE4, cardiovascular diseases, therapy

## Abstract

3′,5′-cyclic adenosine monophosphate (cAMP) is a second messenger critically involved in the control of a myriad of processes with significant implications for vascular and cardiac cell function. The temporal and spatial compartmentalization of cAMP is governed by the activity of phosphodiesterases (PDEs), a superfamily of enzymes responsible for the hydrolysis of cyclic nucleotides. Through the fine-tuning of cAMP signaling, PDE4 enzymes could play an important role in cardiac hypertrophy and arrhythmogenesis, while it decisively influences vascular homeostasis through the control of vascular smooth muscle cell proliferation, migration, differentiation and contraction, as well as regulating endothelial permeability, angiogenesis, monocyte/macrophage activation and cardiomyocyte function. This review summarizes the current knowledge and recent advances in understanding the contribution of the PDE4 subfamily to cardiovascular function and underscores the intricate challenges associated with targeting PDE4 enzymes as a therapeutic strategy for the management of cardiovascular diseases.

## 1. Introduction

Cyclic nucleotide phosphodiesterases (PDEs) comprise a superfamily of evolutionary highly conserved enzymes catalyzing the hydrolysis of cyclic adenosine monophosphate (cAMP) and cyclic guanosine monophosphate (cGMP), second messengers in multiple physiological and pathological processes affecting cardiac and vascular function, such as ionotropic and chronotropic responses and vascular cell proliferation and migration, among others [1,2,3].

The activity of PDEs tightly regulates the magnitude and duration of cAMP and cGMP signaling, which, in turn, impacts physiologic and metabolic processes, including inflammation, ion channel-dependent signaling, cell apoptosis and differentiation, muscle contraction, lipogenesis, glycogen synthesis and gluconeogenesis [4]. Notably, the disturbance of the activity or expression of these enzymes has been linked to a vast array of disorders, including cancer and neurological, respiratory and cardiovascular diseases [2,3], relevant disorders that have a major impact on people’s health and significantly contribute to the economic burden worldwide.

The PDE superfamily is composed of 11 families of enzymes (PDE1 to PDE11), grouped according to their homology within the C-terminal catalytic domain. These enzymes exhibit different substrate selectivities. Specifically, PDE4, PDE7 and PDE8 selectively degrade cAMP, while PDE5, PDE6 and PDE9 are selective for cGMP, and PDE1, PDE2, PDE3, PDE10 and PDE11 can hydrolyze both substrates. Several isoforms are recognized within each family, distinguished by the length and complexity of the N-terminal regulatory domain, which confers distinct kinetic properties [1,2,3,5]. PDEs not only modulate the cellular content of cyclic nucleotides but, more importantly, also contribute to their subcellular compartmentalization. This circumstance gives rise to temporal and spatial gradients of these secondary messengers, thereby regulating their cellular effects in time and space. Each tissue and cell type expresses different PDEs, and although they may be somewhat redundant, it is now widely recognized that each PDE variant carries out specific physiological functions [5,6,7]. Consequently, this family of enzymes has emerged as a promising pharmacological target. In the context of the cardiovascular system, alterations in intracellular levels of cAMP and cGMP regulate the contraction/relaxation, proliferation, migration and response to damage of vascular smooth muscle cells (VSMCs), as well as modulating cardiomyocyte contraction and endothelial function [3,6,8].

**Figure 1:**
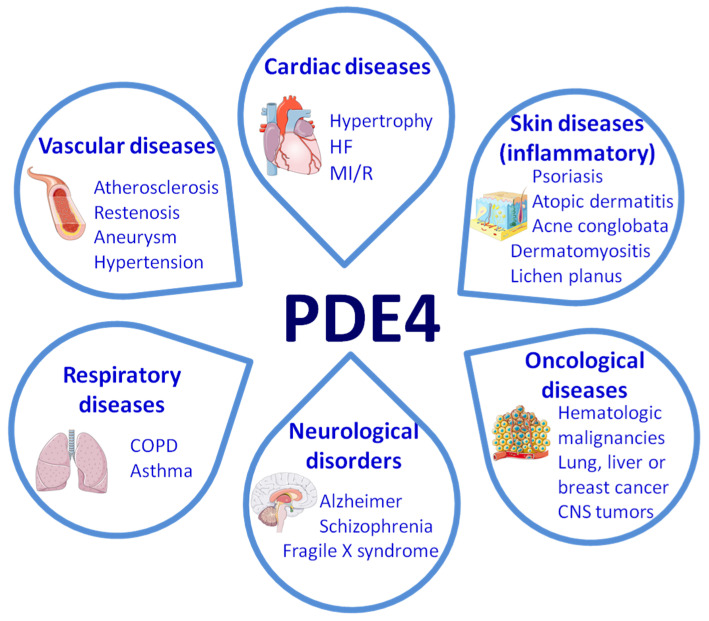
PDE4 in disease. This scheme depicts the diseases that have been associated with disturbances in PDE4 expression/activity. HF: Heart failure; MI/R myocardial ischemia/reperfusion; COPD: chronic obstructive pulmonary disease; CNS: central nervous system. This figure was partly generated using Servier Medical Art provided by Servier, licenced under a Creative Commons Attribution 3.0 unported license.

The members of the PDE4 subfamily represent the main cAMP-selective PDEs in different organs and cell types and are characterized by their sensitivity to rolipram inhibition. This subfamily, which is one of the most studied, comprises four members with multiple variants that are expressed in a wide range of tissues and have been implicated in numerous pathophysiological processes. By controlling the degradation rate of cAMP, the PDE4 subfamily plays a fundamental role in the regulation of cardiovascular function [1,2]. This review addresses the contribution of the PDE4 family to biological processes critical for the development of vascular diseases and explores the interest of these enzymes as therapeutic targets (Figure 1).

## 2. The PDE4 Enzyme Family: Structure, Classification and Compartmentalization of cAMP

The PDE4 family is encoded by four genes, *PDE4A*, *PDE4B*, *PDE4C* and *PDE4D*, collectively yielding over 20 different proteins generated by the use of different promoters and by alternative splicing. The catalytic domains of the *PDE4* genes show a 75% sequence homology and a significant structural similarity, rendering the development of selective inhibitors for individual subtypes an ongoing challenge, as mentioned below. Each isoform features a unique N-terminal region. Further, these proteins contain two singular conserved regions, known as Upstream Conserved Regions [UCR] 1 and UCR2, which distinguish them from other PDEs. These UCR regions are located between the N-terminal end and the catalytic region, are considered regulatory regions and determine the subcategorization of PDE4 isoforms. UCR1 and UCR2 are linked to each other by the linker region 1 (LR1), while the LR2 region links UCR2 to the catalytic unit. The LR1 and LR2 regions show high sequence heterogeneity between the different subfamilies, and their function remains essentially unknown [1,9].

**Figure 2:**
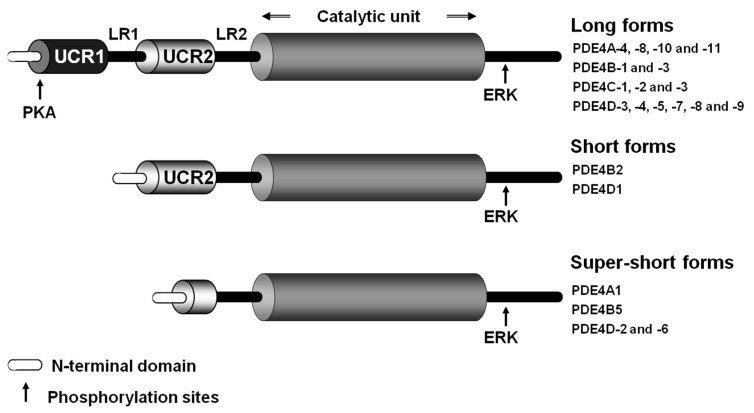
Modular structure of the PDE4 family enzymes. The structure common to the 4 members of the PDE4 family is shown, including UCR1 and UCR2 domains, LR1 and LR2 regions, and the catalytic domain. The isoforms generated by the use of different promoters or by alternative splicing that give rise to the long, short and super-short forms of these enzymes are shown schematically. Phosphorylation sites for PKA and ERK are indicated. The ERK phosphorylation site is not present in PDE4A variants. ERK: Extracellular signal-regulated kinase; LR: linker region; PKA: protein kinase A; UCR: Upstream Conserved Regions.

In general, each of the members of this family presents at least one long isoform comprising the UCR1 and UCR2 regions and, eventually, a short isoform that exclusively contains the UCR2 region. Further, super-short isoforms only partially conserve the UCR2 region (Figure 2). Additionally, transcripts encoding catalytically inactive isoforms (known as dead-short) have been identified. These dead-short isoforms possess a truncated catalytic unit and lack the UCR1 and UCR2 regions; however, they have exclusively been detected at the mRNA level. The UCR1 and UCR2 regions interact with each other and play a fundamental role in the regulation of enzymatic activity. Indeed, in long isoforms, the UCR1–UCR2 interaction causes an autoinhibition of the enzyme. Notably, these two domains facilitate the formation of homo- and heterodimers between long forms, while short forms primarily exist as monomers [10].

The UCR domains can undergo post-translational modification by phosphorylation, affecting PDE function. Indeed, phosphorylation of the UCR1 domain by protein kinase A (PKA) alters the interaction between UCR1 and UCR2 and increases the enzymatic activity of the long isoforms. Since PKA is a direct cAMP effector protein, this modification serves as a negative feedback mechanism to restore cAMP levels through increased PDE4 activity. Furthermore, the presence or absence of UCR1/UCR2 determines the functional consequences of extracellular signal-regulated kinase (ERK)-dependent enzyme phosphorylation, which occurs through the coupling of two motifs located in the catalytic unit and the C-terminal end (phosphorylation site present in all PDE4 subfamilies except PDE4A). Phosphorylation by ERK results in inhibition of long isoforms and activation of short isoforms and leads to a weak inhibition of super-long isoforms [9]. Importantly, PKA-mediated phosphorylation can alter the ERK-induced inhibition of long PDE4 isoforms, enabling a more precise regulation of these enzymes. In addition, phosphorylation may occur in other residues (for example, by Ca^2+^/calmodulin protein kinase II (CAMKII)). Other post-translational modifications, including SUMOylation, also impact enzymatic activity, while transcriptional and epigenetic mechanisms may modulate the expression of specific isoforms [11].

The intracellular localization of PDE4 isoforms is regulated through the N-terminal region. Divergences in the sequence of this region determine the interaction of each isoform with other proteins and its anchoring to specific subcellular compartments, including binding to A-kinase anchoring proteins (AKAPs) [11]. This allows the existence of macromolecular signaling complexes, enabling the specific regulation of pathophysiological processes with therapeutic relevance. For example, the N-terminal region plays a key role, allowing the specific interaction of PDE4A4 and PDE4D4 with the SRC family of tyrosine kinases, the binding of PDE4A4 to hepatitis B virus-associated protein 2 (XAP2), the interaction of PDE4D5 with β-arrestin, the association of PDE4D3 with both muscle-specific AKAP (mAKAP) and AKAP450 and the binding of PDE4D5 with the receptor for activated C kinase 1 (RACK1) [1,12]. The precise localization of PDE4 enzymes is crucial for cAMP compartmentalization and for the interaction with specific proteins that can induce conformational changes, alter the catalytic activity and even influence inhibitors’ affinity. All these mechanisms allow the dynamic regulation of the quantity, localization and activity of PDE4 enzymes to condition and respond to the spatiotemporal modulation of cAMP signaling [11].

PDE4 enzymes are selective for cAMP, with Km values ranging from 1 to 10 µM, and are characterized by their sensitivity to rolipram inhibition [13]. Their broad expression profile encompasses several tissues, including brain, smooth muscle, heart, kidney and endothelial and inflammatory cells, although many of the isoforms and variants show a specific tissue and cellular expression pattern [2,3]. Different studies have suggested the involvement of PDE4 in several pathophysiological processes, supporting the interest of this subfamily of enzymes as potential therapeutic targets for the treatment of a myriad of disorders, including dermatoid arthritis, atopic dermatitis, asthma, chronic obstructive pulmonary disease (COPD), psoriasis, lupus and cardiovascular diseases [2,3,14].

## 3. Role of PDE4 Enzymes in Vascular Cell Function

### 3.1. Role of PDE4 Enzymes in the Endothelium

Vascular endothelium constitutes a selective permeability barrier between blood and tissues that regulates the transport of fluids and macromolecules and plays a critical role in maintaining vascular homeostasis through the control of vascular tone, platelet activation and coagulation, VSMC proliferation, leukocyte trafficking and inflammation. The disturbance of endothelial function (known as endothelial dysfunction) in response to risk factors such as hypercholesterolemia, hypertension or smoking is an early phenomenon in the development of atherosclerosis, involving an imbalance in nitric oxide bioavailability associated with an impaired endothelium-dependent vasorelaxation, induction of proinflammatory factors and altered vascular permeability [15]. Likewise, in sepsis and systemic inflammation, increased microvascular permeability significantly contributes to organ failure and patient death [16].

**Figure 3:**
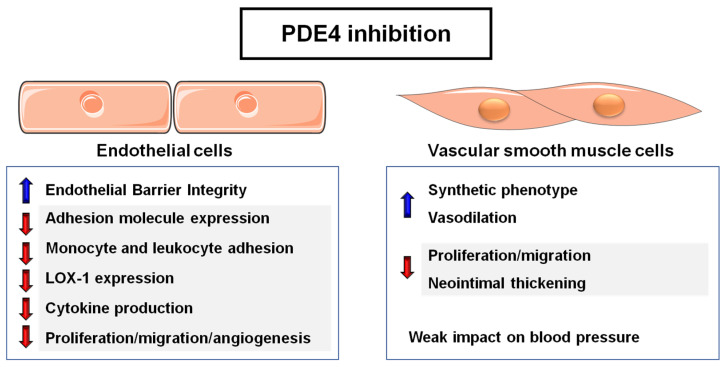
Potential effects of PDE4 inhibition on endothelial cells and vascular smooth muscle cells. LOX-1: Lectin-like oxidized low-density lipoprotein receptor-1.

cAMP is a key player in the stabilization of the endothelial barrier, an aspect particularly relevant in inflammation and sepsis, processes in which the modulation of PDE4 activity holds therapeutic promise. The two main signaling pathways activated by cAMP, PKA and EPAC (Exchange Protein directly Activated by cAMP) lead to the stabilization of the endothelial barrier in a Rac1-dependent manner through the formation of adherens junctions that increase the strength of actin cytoskeleton [17]. In fact, PDE4D, PKA and EPAC1 integrate in a VE–cadherin signaling complex responsible for the control of vascular endothelial permeability [13]. Several studies have evidenced that systemic inflammation is accompanied by a decrease in endothelial cAMP levels and that cAMP signaling is compromised in this pathological scenario [18]. In addition, PDE4 inhibition by rolipram or roflumilast attenuates capillary rupture and improves microcirculation in different rodent models of systemic inflammation, a favorable response arising from the specific PDE4 blockade on endothelial cells [19,20]. PDE4 inhibitors such as roflumilast also decrease leukocyte–endothelial cell interaction in response to lipopolysaccharide (LPS) by preventing the induction of adhesion molecules such as P-selectin and E-selectin [21] (Figure 3). These data highlight the interest in PDE4 inhibition as a relevant therapeutic strategy in this clinical setting.

Likewise, patients with chronic inflammatory diseases such as psoriasis exhibit an increased risk of cardiovascular morbidity and mortality associated with a significant alteration of endothelial function [22]. This underscores the importance of pharmacological strategies addressing both psoriasis and endothelial dysfunction, such as PDE4 inhibition. Studies in human endothelial cells evidenced that apremilast, an inhibitor of PDE4 activity prescribed in psoriatic patients, reduces the expression of oxidized LDL receptor (LOX-1) and inflammatory mediators involved in the development of atherosclerosis, such as tumor necrosis factor alpha (TNF-α), interleukin-6 (IL-6) and IL-8. Further, this drug reduces the adhesion of U937 monocytes to endothelial cells by decreasing the expression of monocyte chemoattractant protein 1 (MCP-1) and vascular cell adhesion molecule 1 (VCAM-1) through a mechanism mediated by the Krüppel-like factor 6 (KLF6) [23]. Similar results were obtained in endothelial cells activated with TNF-α [24], in which, again, apremilast reduced the expression of chemokines and adhesion molecules and limited the adhesion of THP-1 to endothelial cells, ameliorating the endothelial induction of both nuclear factor kappa B (NFĸB) and mitogen-activated protein kinase (MAPK) pathways. These results suggest that apremilast may mitigate the increased cardiovascular risk found in patients with psoriasis (Figure 3) [24].

It is important to highlight the contribution of PDE4 activity to angiogenesis, a process associated with various pathological conditions, including atherosclerosis. In fact, previous studies have shown that the direct activation of PKA by cAMP induces endothelial cell apoptosis and inhibits angiogenesis in vivo [25]. Human endothelial cells express multiple phosphodiesterases, particularly several variants of PDE2, PDE3, PDE4 and PDE5 [2,26,27], although PDE2 and PDE4 are the main enzymes responsible for hydrolyzing cAMP in these cells. In this context, VEGF, a potent angiogenic factor, induces PDE2 and PDE4 activity, as well as the expression of PDE2A, PDE4A, PDE4B and PDE4D. Indeed, in human umbilical endothelial cells (HUVEC) stimulated with VEGF, the combined inhibition of PDE2 and PDE4 activity increases the level of cAMP and decreases proliferation, migration and cell cycle progression. The PDE2 inhibitor reduced S-G2/M phase transition, while the inhibition of PDE4 activity attenuated G0/G1-S transition. These effects were associated with a blockade of cyclins A and D1 expression and an increase in the cell cycle repressors p21 and p27 [28]. Note that the antiangiogenic activity of PDE4 inhibitors has been confirmed in vivo since the blockade of PDE2 and PDE4 limits angiogenesis in the chicken chorioallantoic membrane model [27]. These results emphasize the interest in using PDE2 and PDE4 inhibition to reduce pathological angiogenesis and suggest that the differences observed after PDE2/PDE4 blockade could be attributed to disparities in their subcellular compartmentalization. Similar studies showed that inhibition of PDE4 activity could attenuate inflammatory angiogenesis. Specifically, in mice bearing subcutaneous polyether-polyurethane implants, Mendes et al. demonstrated the effectiveness of rolipram in inhibiting angiogenesis and VEGF production without affecting the accumulation of neutrophils or macrophages [29]. Finally, beyond the cardiovascular field, recent studies have shown that elevated levels of PDE4B in lymphoma cells promote a proangiogenic response, which can be suppressed through genetic or pharmacological inhibition of PDE4 [30]. All these findings highlight the potential of PDE4 inhibition as an antiangiogenic therapeutic strategy in different pathological scenarios (Figure 3).

### 3.2. PDE4 Activity in Vascular Smooth Muscle Cells (VSMCs)

PDE4 activity modulates VSMC proliferation, migration, differentiation, and contraction/relaxation (Figure 3) [6,31,32]. As previously indicated, PDE4 enzymes play a key role in orchestrating the compartmentalization of cAMP-dependent signaling. This is achieved through their anchoring to specific proteins, forming and shaping local cAMP gradients that significantly affect VSMC function and phenotypic regulation [6], as detailed below.

#### 3.2.1. Contribution of PDE4 to the Control of VSMC Proliferation and Migration

Neointimal hyperplasia and luminal stenosis are hallmark processes in several vascular disorders, such as atherosclerosis and restenosis, driven by an exacerbated proliferation and migration of VSMCs to the intima. This results in the thickening of the vascular wall, gradually reducing the arterial lumen [33]. To fight against the restenotic response associated with percutaneous transluminal coronary angioplasty, the main strategy has been the use of covered stents that locally release antiproliferative drugs (sirolimus, paclitaxel and everolimus, among others) [34]. However, this strategy is not free of complications, such as stent thrombosis derived from the concurrent inhibition of re-endothelization. Further, an important limitation is that this approach proves ineffective when dealing with vascular conditions featuring diffuse neointimal lesions. Consequently, it is necessary to identify new therapeutic strategies that limit neointimal thickening in these clinical settings.

In the vascular wall, cAMP inhibits the proliferation and migration of VSMCs both in vitro and in vivo, ultimately limiting neointimal thickening in response to damage [35,36,37]. The regulation of both cAMP-dependent signaling and cell functionality in VSMCs is under the tight control of PDE3 and PDE4 enzymes [38]. Studies in animal models have revealed that percutaneous transluminal coronary angioplasty increases PDE4 expression/activity and decreases cAMP levels [39], potentially contributing to the development of restenosis. Consequently, PDE inhibition may offer therapeutic benefits in this context [39].

Regarding VSMC proliferation, it has been described that the selective inhibition of PDE3, but mainly of PDE4, enhances the antiproliferative effect of adenylate cyclase activators, such as forskolin, which elevate cAMP levels [40]. Similar findings have been obtained for VSMC proliferative and migratory responses [38,41]. These studies have demonstrated that the antimigratory and antiproliferative effects induced by the increase in cAMP levels triggered by forskolin or prostacyclin analogues are exacerbated in the presence of PDE4 inhibitors, while the inhibition of PDE3 does not impact the forskolin antimigratory activity.

#### 3.2.2. VSMC Phenotypic Switch and PDE4 Activity

In healthy arteries, resident VSMCs show a contractile/quiescent phenotype characterized by a low proliferative, migratory and synthetic capacity. However, in response to vascular damage associated with atherosclerosis and restenosis, VSMCs undergo a phenotypic switch to a synthetic/activated phenotype, characterized by a lower contractile capacity and an increase in their migratory, synthetic and proliferative activities. Contractile VSMCs express PDE4A, PDE4B and various variants of PDE4D, the latter being mainly responsible for PDE4 activity in these cells. While both PDE3 and PDE4 are involved in cAMP hydrolysis in VSMCs, the expressed variants and their relative proportions vary depending on cell phenotype, species and vascular bed. Although PDE3 and PDE4 activities are virtually equivalent in contractile cells, in synthetic VSMCs, the drastic decrease in PDE3A expression results in a greater dependence on PDE4 activity for cAMP hydrolysis. In fact, it has been proposed that the ratio between PDE3/PDE4 activities determines the phenotypic differentiation of these cells (Figure 3) [42], with PDE4-dependent signaling playing a relevant role in this process [43]. It should be noted that differences in the PDE3/PDE4 ratio between phenotypes could have therapeutic implications. PDE3 inhibitors would be more effective in relaxing contractile VSMCs, but the reliance on PDE4 activity of synthetic VSMCs would enable the regulation of cAMP-mediated responses specifically in these cells [44]. Actually, although the inhibition of PDE3 activity has been shown to reduce neointimal thickening in preclinical models of restenosis [45], the pro-arrhythmic potential of these drugs makes their clinical use in this context unlikely. In contrast, selective PDE4 inhibitors with modest cardiac effects could offer a safer strategy for reducing post-angioplasty restenosis.

Agents that increase cAMP, such as forskolin, inhibit the proliferation, migration and extracellular matrix synthesis in VSMCs of synthetic phenotype; however, sustained administration of these compounds gives rise to a desensitization phenomenon in which PDE4 activity plays a critical role. The increase in cAMP hydrolysis mediated by PDE4 contributes to this desensitization process in both contractile and synthetic VSMCs through different variants [42]. While the prolonged incubation with cAMP-elevating agents in contractile cells induces the expression of PDE4D3, in synthetic cells, this leads to increased expression of the short variants PDE4D1 and PDE4D2, which are not usually expressed in contractile VSMCs. At the molecular level, it has been shown that the selective induction of PDE4D1 and PDE4D2 in synthetic VSMCs results from increased histone acetylation of an intronic promoter region, a process that regulates the expression of these variants [43]. In turn, these differences could be important from a therapeutic perspective, as PDE4 inhibitors could have a greater impact on cAMP-mediated signaling in synthetic VSMCs, exacerbating the effect of agents that elevate cAMP.

## 4. Role of PDE4 Activity on the Control of Vascular Tone: Impact on Hypertension

Another issue to be considered due to its pathophysiological relevance in the field of vascular diseases such as hypertension and vasospasm is the control of vascular tone that results from the balance between vasoconstrictor and vasodilator signaling pathways. Cyclic nucleotides (cAMP and cGMP) are pivotal modulators of vasodilation under physiological conditions, and therefore, augmenting their levels holds promise as a potential therapeutic approach in cardiovascular diseases. As previously mentioned, in arteries, PDE3 and PDE4 are the primary enzymes responsible for cAMP hydrolysis. Several studies conducted on aortic rings have associated the inhibition of PDE3 activity in VSMCs with vasorelaxation [46,47]. However, unlike PDE3 inhibitors, PDE4 inhibitors such as rolipram generally have a weak hypotensive effect (Figure 3) [48]. Nonetheless, a synergistic vascular relaxation is observed when PDE4 inhibitors are combined with isoproterenol or PDE3 inhibitors [46,49,50]. In fact, in rat coronary arteries, the relaxation induced by the inhibition of PDE3 and PDE4 is controlled by the large-conductance calcium-activated potassium channel (BKCa). Both PDEs colocalize with BKCa channels, allowing strict control of the cAMP available for channel opening, a process that underscores the importance of their subcellular compartmentalization. Certainly, the contribution of this mechanism to the overall regulation of vascular tone by PDEs varies depending on the pathophysiological setting since this coupling, for example, is lost in heart failure (HF) [51].

Despite the limited impact of PDE4 inhibitors on blood pressure, a single-nucleotide polymorphism in the *PDE4D* gene (rs702553) has been associated with blood pressure in the African-American population [52] and, accordingly, recent experimental studies have specifically linked this isoenzyme to hypertension [53]. Indeed, PDE4D expression was increased in the vascular wall of C57BL/6J mice in which hypertension was induced by angiotensin II (Ang II) infusion. In turn, in mice, VSMC-specific knockdown of *PDE4D* attenuated the increase in blood pressure and reduced arterial media thickening, vascular fibrosis, and vasoconstriction triggered by Ang II [53]. Mechanistic studies, both in vitro and in vivo, demonstrated that PDE4D promotes vasoconstriction through the PKA-AMP-activated protein kinase (AMPK)-myosin-specific phosphatase subunit 1 (MYPT1)-myosin light chain (MLC) axis. Of note, treatment of mice with rolipram produced a response similar to that detected in VSMC-specific *PDE4D* knockout mice, resulting in a reduction of the Ang II-mediated increase in blood pressure, medial thickening, vascular fibrosis and vasoconstriction. This suggests the possible usefulness of rolipram in the treatment of hypertension. However, in other experimental models, the impact of PDE4 inhibition on hypertension has yielded conflicting results. In hypertensive rats, neither rolipram nor roflumilast significantly reduced blood pressure, although they did improve the cognitive deficit induced by hypertension in these animals [54,55]. Similarly, the inhibition of PDE4 activity did not significantly modify the increase in blood pressure caused by Ang II in ApoE^−/−^ mice, in which, as described below, the development of aneurysmal lesions was associated with an enhanced vascular expression of PDE4B [56]. These discrepancies may be attributed to differences in the expression pattern and subcellular compartmentalization of these enzymes in each animal model, aspects that have not been thoroughly explored and hamper the clinical translation of research findings. Furthermore, it is important to consider that PDE4 isoenzymes can contribute to the development of hypertension through their activity in other tissues. For instance, the increase in renal PDE4B4 has been linked to salt-sensitive hypertension [57]. Additionally, in the liver, PDE4D would play a key role in the pathogenesis of hypertension associated with non-alcoholic fatty liver disease [58]. For all these reasons and beyond the vascular field, the systemic inhibition of PDE4 would impact multiple processes involved in the mechanisms of hypertension.

## 5. Impact of PDE4 on Vascular Aneurysm Development

Vascular aneurysms are defined as focal dilations of an artery resulting from the weakening of the vascular wall. While these vascular alterations are often asymptomatic, their rupture can lead to severe internal bleeding and high mortality rates. The most common aneurysms include cerebral and aortic aneurysms, with abdominal aortic aneurysms (AAAs) being the most prominent form of this disease. Despite their distinct nature, these disorders share histopathological and biomechanical characteristics, including an exacerbated inflammatory response [59,60,61]. The clinical management of patients with AAA is limited to surgical intervention of large aneurysms, a high-risk surgery with high healthcare costs. Unfortunately, no pharmacological strategies are currently available to limit disease progression and reduce the risk of aneurysm rupture; therefore, a deeper understanding of the pathophysiology of these diseases is essential to identify new therapeutic targets that improve patient care [62].

Due to its ability to regulate cAMP levels, the PDE4 enzyme subfamily could influence key processes involved in aneurysmal diseases, playing an important role in its development and emerging as a potential therapeutic target [63]. Interestingly, PDE4 is considered one of the main therapeutic targets in inflammatory diseases, owing to its critical role in controlling the rate of cAMP degradation in inflammatory and immune cells [64]. However, its relevance in aneurysmal diseases has been poorly explored. Regarding human AAAs, a decrease in the expression of the Gs alpha subunit (GSα), which contributes to the generation of cAMP, has been demonstrated [65]. *GSα* deficiency in VSMCs reduces cAMP production, promotes the phenotypic transition from a contractile to a synthetic state and aggravates the development of AAAs induced by Ang II [65]. Concurrently, in both human and experimental AAAs, our studies demonstrated the increased expression of PDE4B, particularly in the inflammatory infiltrate of the aneurysmal lesion, which would also contribute to a disturbance in vascular cAMP levels [56]. This effect is specific for the B isoform since in AAAs, the expression of PDE4D—the main PDE4 enzyme in VSMCs and the second one in inflammatory cells [6]—remained unaltered. Surprisingly, discrepant data has been recently published, reporting a significant increase in PDE4D expression in both human and experimental AAAs and that the specific deletion of *PDE4D* in VSMCs attenuates aneurysm formation in a mouse model [66]. These inconsistencies might be attributed to differences in the characteristics of each experimental animal model, the heterogeneity of analyzed human AAA lesions and the low number of human samples included in the study conducted by Gao et al. Both studies, however, demonstrated that PDE4 inhibition by rolipram reduced aortic dilation, as well as the incidence and severity of aneurysms, and attenuated the destructive remodeling of the arterial wall, the induction of metalloproteinase activity and the increased vascular oxidative stress induced by Ang II [56,66]. Likewise, and in line with the anti-inflammatory properties described for rolipram [63,67], the inhibition of PDE4 activity reduced vascular inflammation [56]. Notably, although this inhibitor induces vascular relaxation in certain vascular beds [68], rolipram did not affect blood pressure, thus ruling out the influence of hemodynamic responses [56].

Concerning cerebral aneurysms, studies in rats have shown a global increase in the expression of PDE4 isoenzymes. Significantly, and similar to AAAs, the inhibition of PDE4 activity with ibudilast prevented the formation of cerebral aneurysms. This response was associated with a decrease in adhesion molecule expression in the intima and lower recruitment of macrophages in the vascular wall without affecting blood pressure [69].

The blockade of the inflammatory response has demonstrated beneficial effects in limiting aneurysm development in preclinical models [70,71,72]. In this context, the studies described above suggest that suppressing the inflammatory response through the use of PDE4 inhibitors and, more specifically, by targeting PDE4B could constitute a promising strategy. These patients might particularly benefit from the development of new selective PDE4B inhibitors, which are an area of special interest; these molecules might avoid the adverse effects associated with the currently available non-specific PDE4 inhibitors [63].

## 6. PDE4 in Cardiac Diseases

cAMP regulates inotropic and chronotropic responses, impacts cardiomyocyte apoptosis and contributes to cardiac hypertrophy and HF [73]. In this context, PDEs are major drivers defining cAMP levels and compartmentalization in the myocardium, and their inhibition has been proposed as a potential therapeutic intervention to attenuate catecholamine desensitization in HF; however, their chronic use, specifically that of PDE3 inhibitors, has been ruled out due to adverse effects.

In particular, cAMP acts as a second messenger of the sympathetic nervous system in the cardiomyocyte. In fact, the activation of β-adrenergic receptors (β-ARs), which enhance contractile force and heart rate, induces a signaling cascade that promotes changes in cAMP compartmentalization, modulating PKA-dependent phosphorylation of specific target proteins in discrete subcellular organelles of the cardiomyocyte, including ryanodine receptors (RyR2s), sarcolemmal L-type calcium (Ca^2+^) channels (LTCCs), troponin I and phospholamban [74]. This precisely controlled mechanism is dysregulated in cardiac hypertrophy and HF, in which the chronic stimulation of β-ARs induces a detrimental response, leading to maladaptive hypertrophic remodeling, apoptosis and arrhythmias. Of note, the alteration of PDE expression, activity and subcellular localization underlies disease development [73,74,75].

In the heart, the scenario is particularly complex since cardiomyocytes express multiple PDEs with distinct subcellular distribution. PDE3 is abundant in the human heart, in which it associates with the sarcoplasmic reticulum, being the main PDE involved in cAMP hydrolysis and in the control of cardiac contractility [73,75]. Pharmacological interventions with PDE3 inhibitors improve systolic function but, unfortunately, increase the incidence of arrhythmias and enhance morbidity and mortality in patients with HF with reduced ejection fraction [73,76]. Milrinone-treated patients participating in the PROMISE trial had more hospitalizations and hypotension, while little information was provided about the impact of this drug on cardiac parameters [76]. More recently, a study in patients with HF with preserved ejection fraction reported that an extended-release formulation of milrinone improves the quality of life in this group of patients, although no differences in left ventricular size, left ventricular systolic function, and left atrial volume index were found compared with the placebo group [77].

In turn, PDE4 is also expressed in human hearts, although its level is noticeably smaller than that reported in rodents [78], and HF shifts the PDE3/PDE4 balance favoring the PDE3-mediated regulation of β-adrenergic responses. Interestingly, clinical trials in patients with COPD have reported that cardiovascular events were reduced by the PDE4 pan-inhibitor roflumilast [79], supporting the interest in selective PDE4 inhibition for the treatment of cardiac disorders. However, conflicting results have been published in this field.

PDE4 activity is localized to different subcellular compartments. The PDE4D3 variant localizes within the RyR2 complex of the sarcoplasmic reticulum, and its level was decreased in human HF. *PDE4D* inactivation in the RYR2 complex in mice triggers PKA-dependent hyperphosphorylation of the RyR2 [80], which gives rise to a passive calcium leak from the SR and leads to cardiac dysfunction [81] and arrhythmias [82,83], suggesting that PDE4 inhibition could be detrimental in these patients. Similarly, an association between PDE4D and the control of cAMP levels and Ca^2+^ management in the human atrium, both under basal conditions and during β-AR stimulation, has been reported, linking PDE4 activity with a protective response against atrial fibrillation [84]. Furthermore, a single-nucleotide polymorphism (SNP), intronic to *PDE4D*, has been associated with cardiogenic stroke [85], where myocardial diseases are underlying causes. It remains, however, to be determined how the SNP affects PDE4D expression, distribution or activity. PDE4 activity also localizes to SERCA, where a reduction in PDE4 activity might increase Ca^2+^ reuptake, supporting, in this case, a benefit for HF [73]. Further, PDE4 activity is mainly detected in the nuclear envelope. At this location and after the stimulation of β2-ARs, PDE4 hampers the activation of an mAKAPβ-targeted PKA pool, preventing the induction of the pro-apoptotic factor ICER [86] and suggesting that PDE4 inhibition would be deleterious. However, its impact on hypertrophy and HF, in which the β-AR/cAMP/PKA pathway is extensively remodeled, remains to be clarified.

Conversely, a study that compared PDE3- and PDE4-selective inhibitors on inotropic and lusitropic effects showed that PDE4 inhibition by rolipram does not impact the contractile responses to catecholamines in failing human ventricle [87], in agreement with previous data on human ventricular cardiac myocytes [88]. Similarly, we observed that rolipram does not affect Ang II-induced cardiac hypertrophy in hyperlipemic mice [56]. Additionally, it has been reported that HSP20 sequesters PDE4 and that the disruption of this complex led to hyperphosphorylation of HSP20, a protective response that reduces apoptosis and limits the hypertrophic response induced by chronic β-agonist administration. In this context, PDE4 inhibition might increase HSP20 phosphorylation, providing a cardioprotective response [89]. Similarly, recent studies in a mouse model of hypertensive heart disease addressing the mechanisms underlying chronotropic incompetence and sinoatrial node dysfunction in this disorder evidenced that the impaired β-AR regulation of spontaneous action potential firing is mediated by the upregulation of PDE4D [90], data that might have therapeutic repercussions.

While PDE4B is reduced in human HF and myocardial *PDE4B* overexpression attenuates isoprenaline-induced heart hypertrophy/dysfunction [91], suggesting the interest of PDE4B agonists in HF, PDE4B has been proposed as a potential therapeutic target for cardioprotection in patients with acute myocardial infarction subjected to reperfusion therapy [92]. Certainly, cardiac PDE4B expression was specifically upregulated in a mouse model of myocardial ischemia/reperfusion (MI/R) and in patients with coronary artery disease, in which PDE4B was localized in endothelial and myeloid cells. *PDE4B* knockdown studies evidenced that this enzyme plays a critical role in neutrophil activation and microvascular dysfunction after MI/R. Therefore, targeting PDE4B could provide cardioprotection in patients with acute MI referred for reperfusion [92], and PDE4B inhibitors emerge as an appealing pharmacological therapy to ameliorate MI/R injury.

Altogether, these data evidence the complexity of PDE4 signaling in the heart, aggravated by the disparities in the subcellular localization and interaction partners of each variant and the lack of reliable experimental data on established cardiac hypertrophy and HF. It is important to note the discrepancies between mice and humans with regard to PDE expression, compartmentalization and regulation that hinder the translation of experimental insights into the clinics. Further research in this field is, therefore, warranted in order to clarify the interest in targeting PDE4 isoenzymes in cardiac diseases.

## 7. PDE4 Inhibitors: New Approaches

PDE inhibitors emerge as promising therapeutic tools for a wide range of disorders due to their impact on the levels of both cAMP and cGMP and their associated signaling pathways. In this context, a vast array of PDE inhibitors with different selectivities have been developed, although, in practice, the number of PDE-targeting drugs that have reached the market is relatively limited and circumscribed to COPD, inflammatory skin diseases and cardiovascular diseases [93]. Specifically, drugs targeting PDE1, PDE3 and PDE5 enzymes have conclusively evidenced a clinical benefit in the cardiovascular field. This is particularly the case for vinpocetine, a PDE1-specific inhibitor indicated for the management of cerebrovascular disorders. The non-selective PDE3 and PDE5 inhibitor dipyridamole has been approved as an antiplatelet agent used in secondary prophylaxis against postoperative thromboembolic complications [93,94]. In turn, drugs targeting PDE3, such as milrinone, are indicated for the treatment of congestive heart failure despite the increased risk of arrhythmias and hypotension described above. Cilostazol, which is a specific and potent PDE3 inhibitor, is indicated in patients with peripheral arterial disease due to its favorable impact in increasing the walking distance and the quality of life of these patients [93,94]. Further, PDE5-selective inhibitors such as sildenafil and related molecules are particularly valuable, being the first-line therapeutic option in erectile dysfunction.

Besides the clinical application of the PDE inhibitors described above, preclinical studies support that PDE4 enzymes, which constitute the greatest PDE family with four members and multiple variants, critically control vascular and cardiac function by fine-tuning cAMP-dependent phosphorylation cascades (Table 1); thereby, these enzymes also constitute an appealing therapeutic target in cardiovascular diseases. However, no PDE4 inhibitor is currently indicated for the treatment of these disorders.

Further, despite the efforts in the development of selective PDE4 inhibitors, the clinical application of the currently available compounds and their market access have been hindered by the occurrence of serious side effects, including headache, dizziness and gastrointestinal disorders (nausea, vomiting, diarrhea and abdominal pain), which have been linked to the nonspecific blockade of PDE4D enzymes [4]. This has limited the number of PDE4 inhibitors currently on the market to four (Table 2). These are roflumilast, apremilast, crisaborole and drotaverine, indicated for the treatment of COPD, psoriasis, atopic dermatitis and pain due to smooth muscle spasms in biliary and urinary diseases, respectively (Table 2). Notably, two additional inhibitors are in phase II clinical trials—RPL554, for the treatment of COPD, and BPN14770, for the management of patients with Alzheimer’s disease and fragile X syndrome [11].

The severity of the adverse effects of these drugs has evidenced the need for more specific inhibitors to improve both the efficacy and safety of the treatment. Successful therapeutic strategies targeting PDEs must go beyond the mere blockade of the enzyme’s catalytic site. The wide diversity of isoforms, each of them with a unique tissue expression profile, subcellular compartmentalization, conformational state and protein–protein interaction, is a challenge for the development of selective therapeutic strategies that restore cAMP-dependent signaling in the affected compartments without altering signaling in other regions, achieving a maximum therapeutic efficacy while ensuring safety. This requires a deeper understanding of the biology and structure of each of the isoforms and how the subcellular compartmentalization of cAMP is disturbed in each disease. Of note, most of the available data regarding the contribution of PDE4 to pathophysiological processes come from studies performed with non-selective PDE4 inhibitors and do not have a clear picture of the subcellular signaling complexes involved.

The design of selective PDE inhibitors based on the blockade of the catalytic site has been hampered by the high structural and conformational similarities in the catalytic domain, an issue particularly relevant for PDE4B and PDE4D. This has boosted alternative strategies, such as the development of negative allosteric regulators instead of direct catalytic inhibitors. The discrepancies in the amino acid sequence of the UCR2 region between PDE4D and the other members of this subfamily have allowed the design of selective allosteric inhibitors for PDE4D, such as BPN14770, mentioned above and currently in clinical trials (Table 2) [96]. Innovative strategies in this field include the use of gene silencing therapy for the specific inhibition of certain isoforms, the use of catalytically inactive dominant negatives that compete with the endogenous isoforms for protein partners or the use of small molecules or peptides to modulate the interaction of a specific PDE4 isoform with a particular protein, allowing for the precise regulation of signaling within a given compartment [97]. For instance, a peptide targeting the binding region of PDE4 with HSP20 has demonstrated its ability to increase the PKA-dependent phosphorylation of this heat-shock protein, avoiding the hypertrophic response induced by β-AR stimulation [89]. Likewise, new release systems are being studied to avoid the problems associated with the systemic administration of these compounds. In this context, there is a growing interest in the use of nanovesicles as cilomilast carriers, reducing its adverse effects on the nervous system [98]. At the same time, efforts are being made towards achieving selective PDE4 inhibition within the immune compartment. This approach involves the use of antibodies against CD11a conjugated with a potent inhibitor of PDE4 activity, GSK256066, demonstrating the feasibility of the administration of these drugs in a tissue-specific manner [95].

## 8. Conclusions and Future Directions

Despite the potential therapeutic benefit in preclinical research, currently available PDE4 inhibitors are not indicated for the treatment of cardiovascular diseases. However, a meta-analysis of clinical trials in patients with COPD receiving roflumilast has revealed that treatment with this PDE4 inhibitor is associated with a significant reduction in major cardiovascular events [79]. This response may be linked to improved endothelial function and VSMC functionality but also to the inhibition of leukocyte–platelet interaction and the attenuation of the prothrombotic activity of polymorphonuclear leukocytes and monocytes [99]. Of note, while most research has been focused on long PDE4 isoforms, short isoforms have recently aroused growing interest [100]. It becomes evident that the development of more effective and safer PDE4 inhibitors requires a deep knowledge of their selectivity for specific subtypes, isoforms, conformations and subcellular locations, increasing the chance of success and the potential clinical application of these drugs.

## Figures and Tables

**Table 1:** Preclinical studies targeting PDE4 enzymes.

Animal/Cellular Model	Type of Study	Strategy of PDE4 Inhibition	Impact	Ref
Rats	in vivo	Rolipram/roflumilast	Endothelial barrier stabilization	[19]
Rats	in vivo	rolipram	Improved endothelial barrier	[20]
Rats/HUVEC	in vivo/vitro	roflumilast	Reduced leukocyte adhesion	[21]
HAEC	in vitro	apremilast	Reduced monocyte adhesion and inflammatory mediators	[23]
HUVEC	in vitro	apremilast	Attenuated monocyte adhesion and inflammation	[24]
CAM model/HUVEC	in vivo/vitro	RP73401/RP73402	Reduced angiogenesis	[27]
HUVEC	in vitro	RP73402	Decreased proliferation/cell cycle progression	[28]
Mice	in vivo	rolipram	Decreased angiogenesis	[29]
DLBCL cell lines	in vitro	roflumilast	Decreased angiogenesis	[30]
VSMCs	in vitro	Ro-20-1724	Antiproliferative	[38]
VSMCs	in vitro	Ro-20-1724/rolipram	Antiproliferative	[40]
PASMC	in vitro	Roflumilast/rolipram/ cilomilast	Antiproliferative, MMP2/MMP9 reduction	[41]
Rats	in vivo	Ro-20-1724/zardaverine	Vascular relaxation	[42]
Rats/VSMCs	in vivo/vitro	Ro-20-1724	Normalization of cAMP function	[43]
VSMCs	in vitro	Ro-20-1724	Vascular relaxation	[49]
Rat aorta	ex vivo	rolipram	Vascular relaxation	[50]
Coronary arteries	ex vivo	Ro-20-1724	Vascular relaxation	[51]
Ang II-infused mice/MRAs/VSMCs	in vivo/vitro, ex vivo	Rolipram/*PDE4D* knockdown	Antihypertensive, reduced vascular fibrosis, thickening and vasoconstriction	[53]
Hypertensive rats	in vivo	rolipram, roflumilast	Improved hypertension-induced cognitive deficit	[54]
Hypertensive rats	in vivo	rolipram, roflumilast	Memory improvement in hypertension	[55]
ApoE^−/−^ mice	in vivo	rolipram	Inhibition of AAA	[56]
HFD-fed mice	in vivo/vitro	roflumilast	Attenuated hepatic steatosis and dyslipidemia	[58]
ApoE^−/−^ mice	in vivo	Roflumilast/*PDE4D* knockdown	Inhibition of AAA	[66]
Aortic rings	ex vivo	denbufylline, rolipram	Vascular relaxation	[68]
Rats	in vivo	Ibudilast	Inhibition of cerebral aneurysm	[69]
PDE4D^−/−^ mice	in vivo	*PDE4D* knockdown	Heart failure and arrhythmias	[80]
Myocytes under β_2_-AR stimulation	in vitro	Ro-201724	Potentiation of PKA activity	[86]
Human LV trabeculae	in vitro	Rolipram	No impact on catecholamine-induced lusitropic and inotropic effects	[87]
Cardiac myocytes/HEKB2 cells	in vitro	Rolipram	Attenuation of β-agonist-induced hypertrophy	[89]
Ang II-infused mice	in vivo	Rolipram/*PDE4D* knockdown	PDE4D impairs β-adrenergic signaling	[90]
Transgenic mice	in vivo	*PDE4B* transgenesis	Cardioprotection	[91]
PDE4B^−/− ^mice	in vivo	*PDE4B* knockdown	Protection against MI/R	[92]
Human monocytes	in vitro	GSK256066 conjugated with αCD11a	Suppression of LPS-induced TNF-α secretion	[95]

AAA: abdominal aortic aneurysm; Ang II: angiotensin II; ApoE: apolipoprotein E; AR: adrenergic receptor; CAM: chorioallantoic membrane assay; DLBCL: Diffuse large B-cell non-Hodgkin′s lymphoma; HAEC: human aortic endothelial cells; HFD: high-fat diet; HUVEC: human umbilical vein endothelial cells; LV: left ventricle; LPS: lipopolysaccharide; MMP: matrix metalloproteinase; MI/R: myocardial ischemia/reperfusion; MRA: mesenteric artery; PASMC: pulmonary artery smooth muscle cells; PDE4: phosphodiesterase 4; Ref: Reference; TNF: tumor necrosis factor; VSMCs: vascular smooth muscle cells.

**Table 2:** PDE4 inhibitors in clinical practice or in clinical trials.

Name (Agency)	Therapeutic Use	Status	Clinical Trial *	Side-Effects
**Roflumilast** (EMEA/FDA)	COPD Asthma Bronchiectasis	Approved Phase II Phase II	NCT03532490 NCT03988816	Diarrhea, weight and appetite loss, nausea, headache, insomnia
**Apremilast** (EMEA/FDA)	Psoriatic arthritis Acne conglobata Dermatomyositis Vulvar lichen planus Behçet syndrome Psoriasis Alcohol use disorders	Approved Phase II Phase II Phase II Phase III Phase IV Phase II	NCT04161456 NCT03529955 NCT03656666 NCT02307513 NCT03022617 NCT03175549	Diarrhea, vomiting, weight loss and mood swings
**Crisaborole** (EMEA #/FDA)	Atopic dermatitis Morphea Seborrheic dermatitis	Approved Phase II Phase IV	NCT03351114 NCT03567980	Dermal hypersensitivity
**Drotaverine** (Ro, Sk, Hu, Ch) †	Spasmodic pain in biliary disease	Approved		Fainting, nausea, vomiting and dry mouth
**RPL554**	COPD	Phase II	NCT03937479	
**BPN14770**	Alzheimer’s disease Fragile X syndrome	Phase II Phase II	NCT03817684 NCT03569631	

COPD: Chronic obstructive pulmonary disease. Ch: Switzerland; Hu: Hungary; Ro: Romania; Sk: Slovakia. * The National Clinical Trial (NCT) number assigned by ClinicalTrials.gov is indicated. # Concerns have been raised by EMEA about pediatric use. † Not approved by FDA/EMEA.

## References

[1] Omori K., Kotera J. (2007). Overview of PDEs and their regulation. Circ. Res..

[2] Keravis T., Lugnier C. (2012). Cyclic nucleotide phosphodiesterase (PDE) isozymes as targets of the intracellular signalling network: Benefits of PDE inhibitors in various diseases and perspectives for future therapeutic developments. Br. J. Pharmacol..

[3] Bender A.T., Beavo J.A. (2006). Cyclic nucleotide phosphodiesterases: Molecular regulation to clinical use. Pharmacol. Rev..

[4] Peng T., Qi B., He J., Ke H., Shi J. (2020). Advances in the development of phosphodiesterase-4 inhibitors. J. Med. Chem..

[5] Francis S.H., Blount M.A., Corbin J.D. (2011). Mammalian cyclic nucleotide phosphodiesterases: Molecular mechanisms and physiological functions. Physiol. Rev..

[6] Houslay M.D., Baillie G.S., Maurice D.H. (2007). cAMP-Specific phosphodiesterase-4 enzymes in the cardiovascular system: A molecular toolbox for generating compartmentalized cAMP signaling. Circ. Res..

[7] Lugnier C. (2006). Cyclic nucleotide phosphodiesterase (PDE) superfamily: A new target for the development of specific therapeutic agents. Pharmacol. Ther..

[8] Zaccolo M., Movsesian M.A. (2007). cAMP and cGMP signaling cross-talk: Role of phosphodiesterases and implications for cardiac pathophysiology. Circ. Res..

[9] Schick M.A., Schlegel N. (2022). Clinical implication of phosphodiesterase-4-inhibition. Int. J. Mol. Sci..

[10] Bolger G.B., Dunlop A.J., Meng D., Day J.P., Klussmann E., Baillie G.S., Adams D.R., Houslay M.D. (2015). Dimerization of cAMP phosphodiesterase-4 (PDE4) in living cells requires interfaces located in both the UCR1 and catalytic unit domains. Cell Signal..

[11] Paes D., Schepers M., Rombaut B., van den Hove D., Vanmierlo T., Prickaerts J. (2021). The molecular biology of phosphodiesterase 4 enzymes as pharmacological targets: An interplay of isoforms, conformational states, and inhibitors. Pharmacol. Rev..

[12] Huston E., Houslay T.M., Baillie G.S., Houslay M.D. (2006). cAMP phosphodiesterase-4A1 (PDE4A1) has provided the paradigm for the intracellular targeting of phosphodiesterases, a process that underpins compartmentalized cAMP signalling. Biochem. Soc. Trans..

[13] Lugnier C. (2022). The complexity and multiplicity of the specific cAMP phosphodiesterase family: PDE4, open new adapted therapeutic approaches. Int. J. Mol. Sci..

[14] Bobin P., Belacel-Ouari M., Bedioune I., Zhang L., Leroy J., Leblais V., Fischmeiste R., Vandecasteele G. (2016). Cyclic nucleotide phosphodiesterases in heart and vessels: A therapeutic perspective. Arch. Cardiovasc. Dis..

[15] Peng Z., Shu B., Zhang Y., Wang M. (2019). Endothelial Response to Pathophysiological Stress. Arterioscler. Thromb. Vasc. Biol..

[16] Bermejo-Martin J.F., Martín-Fernandez M., López-Mestanza C., Duque P., Almansa R. (2018). Shared features of endothelial dysfunction between sepsis and its preceding risk factors (aging and chronic disease). J. Clin. Med..

[17] Schlegel N., Waschke J. (2014). cAMP with other signaling cues converges on Rac1 to stabilize the endothelial barrier—A signaling pathway compromised in inflammation. Cell Tissue Res..

[18] Schlegel N., Baumer Y., Drenckhahn D., Waschke J. (2009). Lipopolysaccharide-induced endothelial barrier breakdown is cyclic adenosine monophosphate dependent in vivo and in vitro. Crit. Care Med..

[19] Schick M.A., Wunder C., Wollborn J., Roewer N., Waschke J., Germer C.T., Schlegel N. (2012). Phosphodiesterase-4 inhibition as a therapeutic approach to treat capillary leakage in systemic inflammation. J. Physiol..

[20] Flemming S., Schlegel N., Wunder C., Meir M., Baar W., Wollborn J., Roewer N., Germer C.T., Schick M.A. (2014). Phosphodiesterase 4 inhibition dose dependently stabilizes microvascular barrier functions and microcirculation in a rodent model of polymicrobial sepsis. Shock.

[21] Sanz M.J., Cortijo J., Taha M.A., Cerdá-Nicolás M., Schatton E., Burgbacher B., Klar J., Tenor H., Schudt C., Issekutz A.C. (2007). Roflumilast inhibits leukocyte-endothelial cell interactions, expression of adhesion molecules and microvascular permeability. Br. J. Pharmacol..

[22] Brezinski E.A., Follansbee M.R., Armstrong E.J., Armstrong A.W. (2014). Endothelial dysfunction and the effects of TNF inhibitors on the endothelium in psoriasis and psoriatic arthritis: A systematic review. Curr. Pharm. Des..

[23] Wang H., Yang G., Zhang Q., Liang X., Liu Y., Gao M., Guo Y., Chen L. (2020). Apremilast ameliorates ox-LDL-induced endothelial dysfunction mediated by KLF6. Aging.

[24] Otto M., Dorn B., Grasmik T., Doll M., Meissner M., Jakob T., Hrgovic I. (2022). Apremilast effectively inhibits TNFα-induced vascular inflammation in human endothelial cells. J. Eur. Acad. Venereol..

[25] Kim S., Bakre M., Yin H., Varner J.A. (2002). Inhibition of endothelial cell survival and angiogenesis by protein kinase A. J. Clin. Investig..

[26] Netherton S.J., Maurice D.H. (2005). Vascular endothelial cell cyclic nucleotide phosphodiesterases and regulated cell migration: Implications in angiogenesis. Mol. Pharmacol..

[27] Favot L., Keravis T., Holl V., Le Bec A., Lugnier C. (2003). VEGF-induced HUVEC migration and proliferation are decreased by PDE2 and PDE4 inhibitors. Thromb. Haemost..

[28] Favot L., Keravis T., Lugnier C. (2004). Modulation of VEGF-induced endothelial cell cycle protein expression through cyclic AMP hydrolysis by PDE2 and PDE4. Thromb. Haemost..

[29] Mendes J.B., Rocha M.A., Araújo F.A., Moura S.A., Ferreira M.A., Andrade S.P. (2009). Differential effects of rolipram on chronic subcutaneous inflammatory angiogenesis and on peritoneal adhesion in mice. Microvasc. Res..

[30] Suhasini A.N., Wang L., Holder K.N., Lin A.P., Bhatnagar H., Kim S.W., Moritz A.W., Aguiar R.C.T. (2016). A phosphodiesterase 4B-dependent interplay between tumor cells and the microenvironment regulates angiogenesis in B-cell lymphoma. Leukemia.

[31] Chen W.J., Chen Y.H., Lin K.H., Ting C.H., Yeh Y.H. (2011). Cilostazol promotes vascular smooth muscles cell differentiation through the cAMP response element-binding protein-dependent pathway. Arterioscler. Thromb. Vasc. Biol..

[32] Fetalvero K.M., Shyu M., Nomikos A.P., Chiu Y.F., Wagner R.J., Powell R.J., Hwa J., Martin K.A. (2006). The prostacyclin receptor induces human vascular smooth muscle cell differentiation via the protein kinase A pathway. Am. J. Physiol. Heart Circ. Physiol..

[33] Wadey K., Lopes J., Bendeck M., George S. (2018). Role of smooth muscle cells in coronary artery bypass grafting failure. Cardiovasc. Res..

[34] Onuma Y., Serruys P.W. (2011). Bioresorbable scaffold: The advent of a new era in percutaneous coronary and peripheral revascularization?. Circulation.

[35] Indolfi C., Avvedimento E.V., Di Lorenzo E., Esposito G., Rapacciuolo A., Giuliano P., Grieco D., Cavuto L., Stingone A.M., Ciullo I. (1997). Activation of cAMP-PKA signaling in vivo inhibits smooth muscle cell proliferation induced by vascular injury. Nat. Med..

[36] McKean J.S., Murray F., Gibson G., Shewan D.A., Tucker S.J., Nixon G.F. (2015). The cAMP-producing agonist beraprost inhibits human vascular smooth muscle cell migration via exchange protein directly activated by cAMP. Cardiovasc. Res..

[37] Wu Y.J., Bond M., Sala-Newby G.B., Newby A.C. (2006). Altered S-phase kinase-associated protein-2 levels are a major mediator of cyclic nucleotide-induced inhibition of vascular smooth muscle cell proliferation. Circ. Res..

[38] Palmer D., Tsoi K., Maurice D.H. (1998). Synergistic inhibition of vascular smooth muscle cell migration by phosphodiesterase 3 and phosphodiesterase 4 inhibitors. Circ. Res..

[39] Zhao H., Guan Q., Smith C.J., Quilley J. (2008). Increased phosphodiesterase 3A/4B expression after angioplasty and the effect on VASP phosphorylation. Eur. J. Pharmacol..

[40] Souness J.E., Hassall G.A., Parrott D.P. (1992). Inhibition of pig aortic smooth muscle cell DNA synthesis by selective type III and type IV cyclic AMP phosphodiesterase inhibitors. Biochem. Pharmacol..

[41] Growcott E.J., Spink K.G., Ren X., Afzal S., Banner K.H., Wharton J. (2006). Phosphodiesterase type 4 expression and anti-proliferative effects in human pulmonary artery smooth muscle cells. Respir. Res..

[42] Tilley D.G., Maurice D.H. (2002). Vascular smooth muscle cell phosphodiesterase (PDE) 3 and PDE4 activities and levels are regulated by cyclic AMP in vivo. Mol. Pharmacol..

[43] Tilley D.G., Maurice D.H. (2005). Vascular smooth muscle cell phenotype-dependent phosphodiesterase 4D short form expression: Role of differential histone acetylation on cAMP-regulated function. Mol. Pharmacol..

[44] Maurice D.H., Palmer D., Tilley D.G., Dunkerley H.A., Netherton S.J., Raymond D.R., Elbatarny H.S., Jimmo S.L. (2003). Cyclic nucleotide phosphodiesterase activity, expression, and targeting in cells of the cardiovascular system. Mol. Pharmacol..

[45] Smith S.A., Newby A.C., Bond M. (2019). Ending restenosis: Inhibition of vascular smooth muscle cell proliferation by cAMP. Cells..

[46] Lugnier C., Komas N. (1993). Modulation of vascular cyclic nucleotide phosphodiesterases by cyclic GMP: Role in vasodilatation. Eur. Heart J..

[47] Kauffman R.F., Schenck K.W., Utterback B.G., Crowe V.G., Cohen M.L. (1987). In vitro vascular relaxation by new inotropic agents: Relationship to phosphodiesterase inhibition and cyclic nucleotides. J. Pharmacol. Exp. Ther..

[48] Polson J.B., Strada S.J. (1996). Cyclic nucleotide phosphodiesterases and vascular smooth muscle. Annu. Rev. Pharmacol. Toxicol..

[49] Maurice D.H., Crankshaw D., Haslam R.J. (1991). Synergistic actions of nitrovasodilators and isoprenaline on rat aortic smooth muscle. Eur. J. Pharmacol..

[50] Lindgren S.H., Andersson T.L., Vinge E., Andersson K.E. (1990). Effects of isozyme-selective phosphodiesterase inhibitors on rat aorta and human platelets: Smooth muscle tone, platelet aggregation and cAMP levels. Acta Physiol. Scand..

[51] Idres S., Perrin G., Domergue V., Lefebvre F., Gomez S., Varin A., Fischmeister R., Leblais V., Manoury B. (2019). Contribution of BKCa channels to vascular tone regulation by PDE3 and PDE4 is lost in heart failure. Cardiovasc. Res..

[52] Anthony E.G., Richard E., Lipkowitz M.S., Kelley S.T., Alcaraz J.E., Shaffer R.A., Bhatnagar V. (2013). Association of phosphodiesterase 4 polymorphism (rs702553) with blood pressure in the African American Study of Kidney Disease and Hypertension Genomics Study. Pharmacogenet. Genom..

[53] Fan T., Hou Y., Ge W., Fan T., Feng X., Guo W., Song X., Gao R., Wang J. (2022). Phosphodiesterase 4D promotes angiotensin II-induced hypertension in mice via smooth muscle cell contraction. Commun. Biol..

[54] Jabaris S.G., Sumathy H., Kumar R.S., Narayanan S., Thanikachalam S., Babu C.S. (2015). Effects of rolipram and roflumilast, phosphodiesterase-4 inhibitors, on hypertension-induced defects in memory function in rats. Eur. J. Pharmacol..

[55] Jabaris S.S., Sumathy H., Girish R., Narayanan S., Sugumar M., Babu C.S., Thanikachalam S., Thanikachalam M. (2015). Phosphodiesterase-4 inhibitors ameliorates cognitive deficits in deoxycorticosterone acetate induced hypertensive rats via cAMP/CREB signaling system. Brain Res..

[56] Varona S., Puertas L., Galán M., Orriols M., Cañes L., Aguiló S., Camacho M., Sirvent M., Andrés V., Martínez-González J. (2021). Rolipram prevents the formation of abdominal aortic aneurysm (AAA) in Mice: PDE4B as a target in AAA. Antioxidants.

[57] Tawar U., Kotlo K., Jain S., Shukla S., Setty S., Danziger R.S. (2008). Renal phosphodiesterase 4B is activated in the Dahl salt-sensitive rat. Hypertension.

[58] Tao X., He H., Peng J., Xu R., Fu J., Hu Y., Li L., Yang X., Feng X., Zhang C. (2022). Overexpression of PDE4D in mouse liver is sufficient to trigger NAFLD and hypertension in a CD36-TGF-β1 pathway: Therapeutic role of roflumilast. Pharmacol. Res..

[59] Humphrey J.D., Taylor C.A. (2008). Intracranial and abdominal aortic aneurysms: Similarities, differences, and need for a new class of computational models. Annu. Rev. Biomed. Eng..

[60] Sunderland K., Jiang J., Zhao F. (2022). Disturbed flow’s impact on cellular changes indicative of vascular aneurysm initiation, expansion, and rupture: A pathological and methodological review. J. Cell. Physiol..

[61] Torres-Fonseca M., Galan M., Martinez-Lopez D., Cañes L., Roldan-Montero R., Alonso J., Reyero-Postigo T., Orriols M., Mendez-Barbero N., Sirvent M. (2019). Pathophisiology of abdominal aortic aneurysm: Biomarkers and novel therapeutic targets. Clin. Investig. Arterioscler..

[62] Puertas-Umbert L., Almendra-Pegueros R., Jiménez-Altayó F., Sirvent M., Galán M., Martínez-González J., Rodríguez C. (2023). Novel pharmacological approaches in abdominal aortic aneurysm. Clin. Sci..

[63] Azam M.A., Tripuraneni N.S. (2014). Selective phosphodiesterase 4B inhibitors: A review. Sci. Pharm..

[64] Jin J., Mazzacuva F., Crocetti L., Giovannoni M.P., Cilibrizzi A. (2023). PDE4 inhibitors: Profiling hits through the multitude of structural classes. Int. J. Mol. Sci..

[65] Qin X., He L., Tian M., Hu P., Yang J., Lu H., Chen W., Jiang X., Zhang C., Gao J. (2019). Smooth muscle-specific Gsα deletion exaggerates angiotensin II-induced abdominal aortic aneurysm formation in mice in vivo. J. Mol. Cell. Cardiol..

[66] Gao R., Guo W., Fan T., Pang J., Hou Y., Feng X., Li B., Ge W., Fan T., Zhang T. (2022). Phosphodiesterase 4D contributes to angiotensin II-induced abdominal aortic aneurysm through smooth muscle cell apoptosis. Exp. Mol. Med..

[67] Kumar N., Goldminz A.M., Kim N., Gottlieb A.B. (2013). Phosphodiesterase 4-targeted treatments for autoimmune diseases. BMC Med..

[68] Komas N., Lugnier C., Stoclet J.C. (1991). Endothelium-dependent and independent relaxation of the rat aorta by cyclic nucleotide phosphodiesterase inhibitors. Br. J. Pharmacol..

[69] Yagi K., Tada Y., Kitazato K.T., Tamura T., Satomi J., Nagahiro S. (2010). Ibudilast inhibits cerebral aneurysms by down-regulating inflammation-related molecules in the vascular wall of rats. Neurosurgery.

[70] Yuan Z., Lu Y., Wei J., Wu J., Yang J., Cai Z. (2021). Abdominal Aortic Aneurysm: Roles of Inflammatory Cells. Front. Immunol..

[71] Sun J., Sukhova G.K., Yang M., Wolters P.J., MacFarlane L.A., Libby P., Sun C., Zhang Y., Liu J., Ennis T.L. (2007). Mast cells modulate the pathogenesis of elastase-induced abdominal aortic aneurysms in mice. J. Clin. Investig..

[72] Aoki T., Kataoka H., Shimamura M., Nakagami H., Wakayama K., Moriwaki T., Ishibashi R., Nozaki K., Morishita R., Hashimoto N. (2007). NF-kappaB is a key mediator of cerebral aneurysm formation. Circulation.

[73] Kamel R., Leroy J., Vandecasteele G., Fischmeister R. (2023). Cyclic nucleotide phosphodiesterases as therapeutic targets in cardiac hypertrophy and heart failure. Nat. Rev. Cardiol..

[74] Fu Q., Chen X., Xiang Y.K. (2013). Compartmentalization of β-adrenergic signals in cardiomyocytes. Trends Cardiovasc. Med..

[75] Eschenhagen T. (2013). PDE4 in the human heart—Major player or little helper?. Br. J. Pharmacol..

[76] Packer M., Carver J.R., Rodeheffer R.J., Ivanhoe R.J., DiBianco R., Zeldis S.M., Hendrix G.H., Bommer W.J., Elkayam U., Kukin M.L. (1991). Effect of oral milrinone on mortality in severe chronic heart failure. N. Engl. J. Med..

[77] Nanayakkara S., Byrne M., Mak V., Carter K., Dean E., Kaye D.M. (2020). Extended-Release Oral Milrinone for the Treatment of Heart Failure with Pre-served Ejection Fraction. J. Am. Heart Assoc..

[78] Leroy J., Richter W., Mika D., Castro L.R., Abi-Gerges A., Xie M., Scheitrum C., Lefebvre F., Schittl J., Mateo P. (2011). Phosphodiesterase 4B in the cardiac L-type Ca^2+^ channel complex regulates Ca^2+^ current and protects against ventricular arrhythmias in mice. J. Clin. Investig..

[79] White W.B., Cooke G.E., Kowey P.R., Calverley P.M.A., Bredenbröker D., Goehring U.M., Zhu H., Lakkis H., Mosberg H., Rowe P. (2013). Cardiovascular safety in patients receiving roflumilast for the treatment of COPD. Chest.

[80] Lehnart S.E., Wehrens X.H., Reiken S., Warrier S., Belevych A.E., Harvey R.D., Richter W., Jin S.L., Conti M., Marks A.R. (2005). Phosphodiesterase 4D deficiency in the ryanodine-receptor complex promotes heart failure and arrhythmias. Cell.

[81] Reiken S., Gaburjakova M., Gaburjakova J., He K.L., Prieto A., Becker E., Yi G.H., Wang J., Burkhoff D., Marks A.R. (2001). Beta-adrenergic receptor blockers restore cardiac calcium release channel (ryanodine receptor) structure and function in heart failure. Circulation.

[82] Wehrens X.H., Lehnart S.E., Huang F., Vest J.A., Reiken S.R., Mohler P.J., Sun J., Guatimosim S., Song L.S., Rosemblit N. (2003). FKBP12.6 deficiency and defective calcium release channel (ryanodine receptor) function linked to exercise-induced sudden cardiac death. Cell.

[83] Tarifa C., Vallmitjana A., Jiménez-Sábado V., Marchena M., Llach A., Herraiz-Martínez A., Godoy-Marín H., Nolla-Colomer C., Ginel A., Viñolas X. (2022). Spatial Distribution of Calcium Sparks Determines Their Ability to Induce Afterdepolarizations in Human Atrial Myocytes. JACC Basic Transl. Sci..

[84] Molina C.E., Leroy J., Richter W., Xie M., Scheitrum C., Lee I.O., Maack C., Rucker-Martin C., Donzeau-Gouge P., Verde I. (2012). Cyclic adenosine monophosphate phosphodiesterase type 4 protects against atrial arrhythmias. J. Am. Coll. Cardiol..

[85] Gretarsdottir S., Thorleifsson G., Reynisdottir S.T., Manolescu A., Jonsdottir S., Jonsdottir T., Gudmundsdottir T., Bjarnadottir S.M., Einarsson O.B., Gudjonsdottir H.M. (2003). The gene encoding phosphodiesterase 4D confers risk of ischemic stroke. Nat. Genet..

[86] Bedioune I., Lefebvre F., Lechêne P., Varin A., Domergue V., Kapiloff M.S., Fischmeister R., Vandecasteele G. (2018). PDE4 and mAKAPβ are nodal organizers of β2-ARs nuclear PKA signalling in cardiac myocytes. Cardiovasc. Res..

[87] Molenaar P., Christ T., Hussain R.I., Engel A., Berk E., Gillette K.T., Chen L., Galindo-Tovar A., Krobert K.A., Ravens U. (2013). PDE3, but not PDE4, reduces β^1^- and β^2^-adrenoceptor-mediated inotropic and lusitropic effects in failing ventricle from metoprolol-treated patients. Br. J. Pharmacol..

[88] Johnson W.B., Katugampola S., Able S., Napier C., Harding S.E. (2012). Profiling of cAMP and cGMP phosphodiesterases in isolated ventricular cardiomyocytes from human hearts: Comparison with rat and guinea pig. Life Sci..

[89] Sin Y.Y., Edwards H.V., Li X., Day J.P., Christian F., Dunlop A.J., Adams D.R., Zaccolo M., Houslay M.D., Baillie G.S. (2011). Disruption of the cyclic AMP phosphodiesterase-4 (PDE4)-HSP20 complex attenuates the β-agonist induced hypertrophic response in cardiac myocytes. J. Mol. Cell. Cardiol..

[90] Dorey T.W., McRae M.D., Belke D.D., Rose R.A. (2023). PDE4D mediates impaired β-adrenergic receptor signaling in the sinoatrial node in mice with hypertensive heart disease. Cardiovasc. Res..

[91] Karam S., Margaria J.P., Bourcier A., Mika D., Varin A., Bedioune I., Lindner M., Bouadjel K., Dessillons M., Gaudin F. (2020). Cardiac overexpression of PDE4B blunts β-adrenergic response and maladaptive remodeling in heart failure. Circulation.

[92] Wan Q., Xu C., Zhu L., Zhang Y., Peng Z., Chen H., Rao H., Zhang E., Wang H., Chu F. (2022). Targeting PDE4B (phosphodiesterase-4 subtype B) for cardioprotection in acute myocardial infarction via neutrophils and microcirculation. Circ. Res..

[93] Bondarev A.D., Attwood M.M., Jonsson J., Chubarev V.N., Tarasov V.V., Liu W., Schiöth H.B. (2022). Recent developments of phosphodiesterase inhibitors: Clinical trials, emerging indications and novel molecules. Front. Pharmacol..

[94] Gresele P., Momi S., Falcinelli E. (2011). Anti-platelet therapy: Phosphodiesterase inhibitors. Br. J. Clin. Pharmacol..

[95] Yu S., Pearson A.D., Lim R.K., Rodgers D.T., Li S., Parker H.B., Weglarz M., Hampton E.N., Bollong M.J., Shen J. (2016). Targeted delivery of an anti-inflammatory PDE4 inhibitor to immune cells via an antibody-drug conjugate. Mol. Ther..

[96] Berry-Kravis E.M., Harnett M.D., Reines S.A., Reese M.A., Ethridge L.E., Outterson A.H., Michalak C., Furman J., Gurney M.E. (2021). Inhibition of phosphodiesterase-4D in adults with fragile X syndrome: A randomized, placebo-controlled, phase 2 clinical trial. Nat. Med..

[97] Baillie G.S., Tejeda G.S., Kelly M.P. (2019). Therapeutic targeting of 3′,5′-cyclic nucleotide phosphodiesterases: Inhibition and beyond. Nat. Rev. Drug Discov..

[98] Liu F.C., Yu H.P., Lin C.Y., Elzoghby A.O., Hwang T.L., Fang J.Y. (2018). Use of cilomilast-loaded phosphatiosomes to suppress neutrophilic inflammation for attenuating acute lung injury: The effect of nanovesicular surface charge. J. Nanobiotechnol..

[99] Totani L., Amore C., Di Santo A., Dell’Elba G., Piccoli A., Martelli N., Tenor H., Beume R., Evangelista V. (2016). Roflumilast inhibits leukocyte-platelet interactions and prevents the prothrombotic functions of polymorphonuclear leukocytes and monocytes. J. Thromb. Haemost..

[100] Kyurkchieva E., Baillie G.S. (2023). Short PDE4 isoforms as drug targets in disease. Front. Biosci..

